# Periodontal health, xerostomia and oral health-related quality of life in controlled vs. uncontrolled type 2 diabetes mellitus patients

**DOI:** 10.1186/s12903-025-06805-6

**Published:** 2025-08-28

**Authors:** Abhay P. Kolte, Rajashri A. Kolte, Tushar G. Mohata, Pranjali Bawankar, Pavan Bajaj, Mahima Kothekar, Shivani Thakre, Dean

**Affiliations:** 1 Department of Periodontics and Implantology, Ranjeet Deshmukh Dental College and Research Centre, Nagpur, India; 2 Department of Periodontics and Implantology, Sharad Pawar Dental College and Hospital, Datta Meghe Institute of Higher Education and Research (DU), Sawangi (Meghe, Wardha, Meghe India

**Keywords:** Type 2 diabetes mellites, Inflammation, Xerostomia, Oral health related quality of life, OHIP, HbA1C

## Abstract

**Background:**

Diabetes mellitus is a complex endocrine condition affecting the periodontal status of an individual. The current study aimed to assess oral health related quality of life (OHRQL), xerostomia severity, and periodontal status in both controlled and uncontrolled Type 2 Diabetes mellitus. (T2DM)

**Methods:**

Eighty patients with type 2 diabetes aged between 35 and 70 were divided equally in Group I: patients with controlled T2DM and Group II: patients with uncontrolled T2DM. Clinical attachment level (CAL), probing depth (PD), and bleeding on probing (BOP) parameters were assessed. Additionally, xerostomia severity was determined through questionnaire of 11-point xerostomia inventory and OHRQL through 14-point oral health impact profile. (OHIP-14)

**Results:**

The mean HbA1C values differed significantly in the two groups with Group I exhibiting values of 6.97 ± 0.45 gm% and Group II with 9.78 ± 2.31 gm%. The patients in Group II had significantly higher mean PD, CAL, and BOP values in comparisonto Group I. Similar corresponding values were obtained for xerostomia severity and OHIP-14 scores for OHRQL. On comparison between the groups the periodontal status manifested by clinical parameters exhibited more severity while xerostomia severity and OHRQL, was negatively impacted in Group II patients when compared with Group I.

**Conclusion:**

Poor glycaemic control substantially influences periodontal health, xerostomia severity, and oral health-related quality of life. Early periodontal intervention and maintenance of optimal glycaemic control are crucial to improve overall oral and systemic health in diabetic patients.

**Trial registration number:**

CTRI/2025/04/086064 Date: 30 April 2025.

## Background

Diabetes mellitus is fundamentally a multifaceted endocrine disorder that precipitates disturbances in the metabolism of proteins, lipids, and carbohydrates [1]. Because of insulin resistance, the related abnormalities mostly impact tissues like the liver, skeletal muscles, and adipose tissue. The severity and magnitude of the symptoms can differ according and corresponding to time, duration and type of diabetes. Out of the two, Type 2 diabetes mellitus (T2DM) is the more prevalent, affecting 95% of individuals [2, 3]. It is typified by hyperglycaemia effected by deficiencies in either insulin action or secretion, or both. Its prevalence is rising globally as a result of stressful activities and changes in lifestyle, and the resulting mortality is due to impaired renal function and disturbances in the vascular system. Most of the afflicted patients suffer with symptoms such as polydipsia, polyphagia, polyurea, weight loss and vision disorders.

The “sixth complication” of diabetes, as evidenced by comprehensive epidemiological studies, is periodontitis, which is commonly identified in patients with inadequately managed diabetes mellitus compared to those who are healthy. It has been noted that the two chronic illnesses have similar pathologies and risk factors, which show up as inflammation.

The deposition of advanced glycation end products within the tissues and plasma of individuals suffering from T2DM is similarly acknowledged as a trigger for consequent production of inflammatory cytokines, which ultimately leads to the deterioration of periodontal tissues. Furthermore, periodontal diseases may have detrimental effects on metabolic regulation [4, 5]. This correlation is further strengthened by the fact that many studies indicate towards improvement of metabolic control after periodontal therapy, though longitudinal data is desired on this aspect [6, 7].

Apart from periodontal diseases several other intraoral problems are encountered by patients including xerostomia, edentulism, lesions of the soft tissue and ill-fitting appliances which cause considerable discomfort and impact speech, eating patterns, food consumption, digestion, and general health over time [8, 9]. It is postulated that as a consequence to these effects there is a substantial negative influence on the oral health related quality of life (OHRQL) in such individuals. Numerous strategies have been formulated to evaluate the OHRQL, integrating tools such as the Geriatric Oral Health Assessment Index (GOHAI) [10], the Oral Health Impact Profile (OHIP) [11], subjective evaluations of oral health status [12], and the Dental Impact on Daily Performance. The most often used technique for calculating OHRQL’s measure is OHIP, a suitable subjective tool that offers details on how oral conditions have affected a person’s life and how much therapy is thought to be necessary. The OHIP is a survey that measures how individuals perceive the social impact of oral health conditions on their overall health. Slade created the OHIP-14, a condensed version of this with 14 items, in 1997. It demonstrated strong validity, reliability, and accuracy [14].

Preliminary studies indicate that perceptions on the dryness in mouth and subsequent difficulty in the experience of speaking, deglutition, severity of oral diseases affected the OHRQL in patients with xerostomia. While the association between diabetes and periodontitis is well-established, few studies have directly compared the influence of glycaemic control status on periodontal health and xerostomia severity. Thus, this study aimed to evaluate and compare periodontal status, xerostomia severity, and OHRQL in patients with controlled versus uncontrolled Type 2 diabetes mellitus (T2DM).

## Methods

The current investigation was performed from January 2025 to May 2025 after The Institutional Ethics Committee reviewed the protocol and approved it, as it was as per the norms laid down by Helsinki Declaration (1975), revised in 2013 (IEC/RDDC&RC/DEAN/21/2024). Following this the study protocol was duly recorded in the Clinical Trials Registry. The study was implemented after obtaining a written informed consent from every patient who agreed to participate voluntarily with a proper understanding of the purpose and design of it. The sample size was determined using Sample Size Pro version 1.0 software, referring to data from Tabesh et al. [15]. Considering an α error of 0.05, a correlation coefficient between OHIP-14 and xerostomia score of 0.44, a 95% confidence interval, and 80% power, a minimum of 38 patients were required. Accounting for potential dropouts, we included 40 patients per group.

The study encompassed a cohort of 80 patients who were recruited from our institute’s outpatient department specializing in Periodontology and Implantology. The participants who were diagnosed with Stage III Grade B periodontitis based on the recent classification by Tonetti et al. [16]. and aged between 35 and 70 years, were evenly distributed into two groups: Group I, consisting of 40 patients with well-controlled T2DM (HbA1C values ranging from 6.4 to 7.0 gm%), and Group II, comprising 40 patients with poorly controlled T2DM (HbA1C values exceeding 7.0 gm%). The patients who fulfilled the criteria of HbA1C levels, a minimum of eight teeth present in the oral cavity, no history of antibiotic administration within the preceding three months, and no history of periodontal intervention within the prior six months were included. Participants with diagnosed systemic conditions other than T2DM, in addition to pregnant or breastfeeding women, individuals with a history of alcohol dependency, and current smokers were excluded from the study. A detailed case study proforma, inclusive of all recordings related to general and clinical examination findings, was filled for each participant involved. The diabetic status and medication history of all participants were carefully recorded. Patients were under regular medical supervision and were taking oral hypoglycemic agents (OHAs), insulin, or a combination thereof, as prescribed by their physicians. This information was included in the case proforma to account for potential influences on oral health parameters.

Prior to the initiation of the investigation, a sole examiner (TM) employed a UNC-15 periodontal probe (PCPUNC 15; Hu Friedy) to systematically document every intraoral parameter throughout a comprehensive clinical examination of ten patients. The clinical indicators that were meticulously noted Plaque Index (PI) [17], Gingival Index (GI) [18], and Papillary Bleeding Index (PBI) [19]. Prior to periodontal clinical parameters like Probing depth (PD) and clinical attachment levels (CAL) recordings, supragingival scaling was performed to remove gross deposits and ensure accurate probe insertion without altering the subgingival environment or periodontal architecture. This step was crucial to avoid false readings caused by calculus interference and allowed precise measurement of probing depth and clinical attachment level.

Six points around each tooth were measured, and the readings were subsequently adjusted to the nearest 0.5 mm. The same examiner re-recorded these recordings two hours later to conduct an intra-observer repeatability analysis. The intra-class correlation (ICC) coefficient for each periodontal parameter was evaluated through a two-way mixed effects model. With an ICC span of 0.92 to 0.99 among the groups, the intra-observer reliability was classified as excellent.

The severity of xerostomia and its underlying characteristic are represented by a single continuous scale score on the Xerostomia Inventory (XI), a summated rating scale [20]. The 11 XI items have been utilised and validated in numerous investigations to date, addressing both behavioural and experiential elements of the disease [21, 22]. The responses of the patients were recorded in the questionnaire and the subsequent scoring was done. Following the patients’ responses to the questionnaire, a grading process was carried out. The OHIP questionnaire, which has 14 points, was used to collect OHRQL [14]. It is a condensed version of the original long-form comprising of 49 questions, which Locker and Quiñonez reduced to 14 questions and found to have the necessary accuracy [23].

For statistical analysis, IBM Statistics version 20.0 was utilised. Frequency and percentages were calculated for qualitative data, while the mean and standard deviation were determined for quantitative data. PD, CAL, BOP, Xerostomia, and OHIP-14 mean scores were compared between the cohorts utilizing an independent “t” test. To ascertain the interrelationships among various factors, Pearson’s correlation was employed. A significance level of *p* < 0.05 was maintained. The normality of the data was assessed utilizing the Shapiro-Wilk Test, which confirmed that the data adhered to a normal distribution.

## Results

A total of eighty individuals diagnosed with Stage II Grade B periodontitis and T2DM were enrolled and equally divided into two groups based on glycaemic control: Group I (controlled) and Group II (uncontrolled).The age range of participants was 35–70 years, with Group I and Group II having mean ages of 57.40 ± 10.75 years and 54.85 ± 11.42 years, respectively. Overall, there were 48 male and 32 female participants, distributed as 55% male and 45% female in Group I, and 65% male and 35% female in Group II. The mean HbA1C was significantly lower in Group I (6.97 ± 0.45 gm%) compared to Group II (9.78 ± 2.31 gm%). The mean duration of diabetes was comparable between groups (7.59 ± 3.72 years in Group I and 7.56 ± 5.49 years in Group II).

Table 1 summarizes the clinical periodontal parameters, xerostomia severity, and OHIP-14 scores between the two groups. Group II showed significantly higher mean scores in PI, PBI, PD, CAL, BOP, Xerostomia Inventory score, and OHIP-14 score, indicating poorer periodontal health, more severe xerostomia, and lower oral health-related quality of life (OHRQL). Specifically, the mean PI and PBI scores in Group II were 2.22 ± 0.64 and 1.47 ± 0.45, respectively, compared to 1.53 ± 0.56 and 0.89 ± 0.32 in Group I (*p* < 0.05). PD and CAL values were also significantly higher in Group II (3.16 ± 0.73 mm and 5.27 ± 1.32 mm, respectively) than in Group I (2.33 ± 0.79 mm and 3.17 ± 0.93 mm; *p* < 0.001). Group II exhibited higher BOP scores (1.93 ± 0.43; *p* = 0.004), xerostomia scores (35.38 ± 5.15; *p* < 0.001), and OHIP-14 scores (32.05 ± 3.46; *p* < 0.001).

**Table 1:** Descriptive statistics for clinical parameters, Xerostomia severity and OHIP-14 score in two groups

	GROUPS	*N*	Mean (95%CI)	Std. Deviation	*P* VALUE
PI	GROUP I	40	1.5261 (1.02–1.98)	0.5613	< 0.05*
	GROUP II	40	2.2175 (1.97–2.45)	0.6435	
PBI	GROUP I	40	0.8952(0.56–1.13)	0.32	< 0.05*
	GROUP II	40	1.4797(1.13–1.98)	0.45	
PD	GROUP I	40	2.3385 (2.08–2.59)	0.79008	< 0.001
	GROUP II	40	3.1630 (2.86–3.46)	0.72968	
CAL	GROUP I	40	3.1665 (2.86–3.46)	0.93325	< 0.001
	GROUP II	40	5.2788 (4.85–5.70)	1.32126	
BOP	GROUP I	40	1.6353 (1.49–1.77)	0.45232	0.004
	GROUP II	40	1.9272 (1.78–2.06)	0.43120	
XEROSTOMIA	GROUP I	40	24.5750(22.75–26.39)	5.68348	< 0.001
	GROUP II	40	35.3750 (33.72–37.02)	5.14750	
OHIP	GROUP I	40	18.1000 (15.85–20.35)	7.06309	< 0.001
	GROUP II	40	32.0500 (30.94–33.15)	3.45632	
	GROUP II	40	32.0500	3.45632	

Table 2 presents the correlation between xerostomia scores and other study variables. In Group I, xerostomia showed a moderate positive correlation with PD (*r* = 0.44, *p* = 0.04) and OHIP-14 score (*r* = 0.42, *p* = 0.03), while correlations with HbA1C, CAL, and BOP were weak and not statistically significant. In Group II, correlations between xerostomia and all clinical parameters were very weak and did not reach statistical significance.

**Table 2:** Relationship between Xerostomia score and other study variables

		Group I	*p* value	Interpretation
Xerostomia score	HbA1C	0.36	0.24	Weak positive
	PD	0.44	0.04	Moderate positive
	CAL	0.08	0.52	Very weak positive
	BOP	0.27	0.28	Weak positive
	OHIP score	0.42	0.03	Moderate positive

		**Group** **II**	**P ** **value**	**Interpretation**
Xerostomia score	HbA1C	0.19	0.35	Very weak positive
	PD	0.11	0.40	Very weak positive
	CAL	0.08	0.52	Very weak positive
	BOP	0.12	0.42	Very weak positive
	OHIP score	0.18	038	Very weak positive

Table 3 details the correlations between OHIP-14 scores and clinical parameters. In Group I, OHIP-14 score demonstrated a moderate positive correlation with PD (*r* = 0.55, *p* = 0.01) and xerostomia score (*r* = 0.42, *p* = 0.03), and weak positive correlations with HbA1C and BOP. CAL showed a very weak positive correlation. In contrast, Group II showed a weak negative correlation of OHIP-14 score with HbA1C (*r* = − 0.34, *p* = 0.24), and very weak correlations with other variables, indicating less clear associations in patients with poor glycaemic control.

**Table 3:** Relationship between OHIP-14 score and other study variables

		Group I	*P* value	Interpretation
OHIP score	HbA1C	0.30	0.29	Weak positive
	PD	0.55	0.01	Moderate positive
	CAL	0.15	0.41	Very weak positive
	BOP	0.27	0.34	Weak positive
	Xerostomia score	0.42	0.03	Moderate positive
		Group II	P value	Interpretation
OHIP score	HbA1C	−0.34	0.24	Weak negative
	PD	0.05	0.48	Very weak positive
	CAL	−0.04	0.43	Very weak negative
	BOP	−0.20	0.32	Weak negative
	Xerostomia score	0.18	0.53	Very weak positive

Range of Co-relation coefficient – 0.40 to 0.59 – moderate positive; 0.20 to 0.39 – weak positive; 0.00 to 0.19 – very weak positive; −0.39 to −0.20 weak negative; −0.19 to −0.01 very weak negative.

Table 4 depicts the relationships between PI and BOP, xerostomia, and OHIP-14 scores. In Group I, PI exhibited a moderate positive correlation with BOP (*r* = 0.41, *p* = 0.01), and weak positive correlations with xerostomia (*r* = 0.34, *p* = 0.03) and OHIP-14 score (*r* = 0.31, *p* = 0.04). In Group II, PI was moderately correlated with BOP (*r* = 0.48, *p* = 0.002), and showed weak positive correlations with xerostomia (*r* = 0.29, *p* = 0.04) and OHIP-14 score (*r* = 0.36, *p* = 0.02).

**Table 4:** Correlations between PI and BOP, Xerostomia score and OHIP-14 score in both groups

Group	PI Vs BOP (*r*, *p*)	PI Vs Xerostomia (*r*, *p*)	PI Vs OHIP−14 (*r*, *p*)
Group 1	0.41, *p* = 0.01	0.34, *p* = 0.03*	0.31,*p* = 0.04*
Group 2	0.48, *p* = 0.002	0.29,*p* = 0.04*	0.36, *p* = 0.02*

Range of Co-relation coefficient – 0.40 to 0.59 – moderate positive; 0.20 to 0.39 – weak positive; 0.00 to 0.19 – very weak positive; −0.39 to −0.20 weak negative; −0.19 to −0.01 very weak negative.

## Discussion

The currently recognized definition of health emphasizes not only the absence of disease but also a person’s overall physical, mental, and social well-being. Therefore, this evaluation must consider patients’ experiences and perceptions of their condition. Diabetes mellitus is a chronic metabolic disorder that can lead to a wide range of physical symptoms, from mild fatigue to severe complications. With the growing incidence of T2DM and with its capability to influence many other organs of the body, it has affected various systems leading to enormous burden on the health profession. Xerostomia is a common oral condition that can interfere with speaking and chewing, contribute to mucosal irritation and infections, cause atrophic changes in the oral tissues, promote plaque buildup, and diminish the saliva’s natural buffering capacity [24].

In this study, PI and PBI scores were included to provide a measure of oral hygiene status and its potential influence on periodontal inflammation and xerostomia severity. The higher PI and PBI values in Group II suggest poorer plaque control and greater gingival bleeding tendency among patients with uncontrolled diabetes, which likely exacerbates periodontal disease severity and negatively impacts OHRQL. Additionally, the correlations of PI with xerostomia and OHIP-14 scores highlight the role of plaque accumulation in mediating patient-perceived dryness and overall quality of life. These findings underscore the importance of stringent oral hygiene maintenance in diabetic individuals, particularly those with poor metabolic control.

Individuals with T2DM have a higher susceptibility to periodontal disease, characterized by gingival inflammation, formation of periodontal pockets, and clinical attachment loss resulting from progressive bone destruction. The results of the present study confirms this and suggest that the periodontal status as assessed by the clinical parameters indicated the inflammatory changes creeping in to the periodontal tissues. The higher mean PD, CAL and BOP values especially in Group II where the glycaemic status of the patients was uncontrolled indicate increase in severity of these changes. These findings align with previous research suggesting that the accumulation of advanced glycation end products (AGEs) in the tissues and plasma of diabetic patients triggers an exaggerated release of pro-inflammatory cytokines, thereby intensifying inflammation and contributing to periodontal tissue destruction [9, 25]. It has been mentioned that the inflammatory changes are dependent also on the duration of the patient being affected with the disorder. However, in the present study since the duration was almost equal in both the groups so this aspect could not have influenced the results.

An important complaint in diabetic patients is xerostomia, which results in more plaque accumulation causing gingival inflammation and in the presence of decreased ability of host defence mechanism which progresses towards periodontal tissue destruction [8, 26]. The reason ascribed to this has been pointing to the uncontrolled glycaemic status of the individual where the local factors elicit an exaggerated response. As a result, these individuals have more severe periodontal disease and a higher CAL which has been substantiated by the findings of our study. Between Group I and Group II in our study, there was a substantial rise in both mean CAL values and xerostomia scores, indicating that patients with uncontrolled T2DM was suffering from a greater severity of xerostomia which could have been one of the major reasons for the subsequent effects on the clinical status of these patients. It postulates further evidence that T2DM patients especially with uncontrolled glycaemic status can exhibit increased occurrence of periodontitis. Additionally, the duration of diabetes and its metabolic regulation can be important factors in causing xerostomia and hyposalivation, which will further exacerbate the damage.

The OHIP-14 score obtained in the present investigation was 18.10 ± 7.06 in Group I while in Group II it was 32.05 ± 3.45 which is found to be consistent with the previous study though the mean values obtained by us were a bit higher than Tabesh et al. [15] and Verhulst et al. [27]. The probable reason for higher values could be the differential geographical locations, socio economical status and the age of the recruited population. Similar to Tabesh et al. (2023), who reported higher OHIP-14 and xerostomia scores in diabetic patients, our study found significantly worse OHRQL and periodontal parameters in patients with poor glycaemic control. However, our mean OHIP-14 scores were higher, possibly due to differences in population characteristics and oral hygiene practices.

So based on these findings it was inferred that the OHRQL in Group II patients was better than the Group I patients who demonstrated greater OHIP-14 scores. OHRQL has been assessed using the OHIP-14 questionnaire, which has proven to be a reliable tool for evaluating subjective oral health outcomes. The findings of the current study indicate a significant association between OHIP-14 scores and self-perceived severity of xerostomia in diabetic patients, consistent with the results reported by Nikbin et al. [8] and Molania et al. [28]. These findings confirm its value and adaptation to other means of examining the clinical oral status. In fact, a combination of subjective means with the classic objective methods provides better options for reporting of OHRQL.

In Group I patients, the current study shows a somewhat favourable link between PD and xerostomia, suggesting that controlled T2DM would be more acceptable in terms of OHRQL. These findings are different from a few studies where the authors reported a strong correlation between xerostomia and OHRQL, especially its physical domains, in the general population [29, 30]. Since numerous factors possibly impact OHRQL, including general and oral health parameters, formulating treatment models accordingly which will focus on all the aspects is need of the hour. While hyposalivation is a contributing factor in periodontal disease, it does not occur in all patients with uncontrolled T2DM. The presence of glucose in saliva and gingival crevicular fluid provides a rich medium for bacterial proliferation, which, when combined with xerostomia-induced reduced clearance, exacerbates periodontal breakdown.

The findings highlight the critical importance of integrating periodontal care into diabetes management protocols [31]. Close collaboration between dental and medical professionals is essential to ensure comprehensive care, improve glycaemic control, and reduce oral health complications.

The precise impact of xerostomia on OHRQL could not be determined because this study was cross-sectional, and it may have been influenced by confounding variables. Further there are certain limitations of the present study like the absence of a non-diabetic control group, possible cohort heterogeneity, potential confounding factors (e.g., prior smoking, diet, oral hygiene habits), cross-sectional design limitation in establishing causality. More research with a larger sample size longitudinal studies with diverse populations and inclusion of microbiological and salivary biomarker analyses are required to accurately assess how xerostomia or other specific factors affect T2DM patients’ OHRQL in order to enhance their quality of life.

## Conclusion

Within the limitations of the current investigation, it can be said that one of the main causes of inflammatory alterations in the periodontal tissues as shown by PD, CAL, and BOP parameters may be hyposalivation in T2DM patients with inadequate metabolic management. When compared to Group I patients, Group II patients showed greater mean values for xerostomia severity and OHIP-14 scores. These factors cumulatively have influence over the complete oral health and the associated wellbeing thereby affecting OHRQL in such individuals. Early identification and comprehensive periodontal therapy, along with meticulous plaque control, may contribute to improved metabolic control and overall quality of life. Future studies incorporating healthy control groups and longitudinal follow-up are necessary to confirm these associations and clarify causality.

## Data Availability

The datasets generated and/or analysed during the current study are not publicly available due to data privacy issues but are available from the corresponding author on reasonable request.

## References

[1] Jajarm HH, Mohtasham N, Moshaverinia M, Rangiani A. Evaluation of oral mucosa epithelium in type II diabetic patients by an exfoliative cytology method. J Oral Sci. 2008;50(3):335–40. 10.2334/josnusd.50.335. Erratum in: J Oral Sci. 2009;51(3):493. Moshaverinia, Maryam. 18818471 10.2334/josnusd.50.335

[2] Shareef BT, Ang KT, Naik VR. Qualitative and quantitative exfoliative cytology of normal oral mucosa in type 2 diabetic patients. Med Oral Patol Oral Cir Bucal. 2008;13(11):E693–6. 18978708

[3] Report of the Committee on the classification and diagnostic criteria of diabetes mellitus. Diabetes Res Clin Pract. 2002;55(1):65–85. 10.1016/s0168-8227(01)00365-5. 11755481 10.1016/s0168-8227(01)00365-5

[4] Nibali L, Gkranias N, Mainas G, Di Pino A. Periodontitis and implant complications in diabetes. Periodontol 2000. 2022;90(1):88–105. 10.1111/prd.12451. 35913467 10.1111/prd.12451 PMC9805043

[5] Genco RJ, Borgnakke WS. Diabetes as a potential risk for periodontitis: association studies. Periodontol 2000. 2020;83(1):40–5. 10.1111/prd.12270. 32385881 10.1111/prd.12270

[6] Kolte RA, Kolte AP, Bawankar PV, Bajaj VA. Effect of Nonsurgical Periodontal Therapy on Metabolic Control and Systemic Inflammatory Markers in Patients of Type 2 Diabetes Mellitus with Stage III Periodontitis. Contemp Clin Dent. 2023;14(1):45–51. 10.4103/ccd.ccd_514_21. 37249991 10.4103/ccd.ccd_514_21 PMC10209773

[7] Ahuja CR, Kolte AP, Kolte RA, Gupta M, Chari S. Effect of non-surgical periodontal treatment on gingival crevicular fluid and serum leptin levels in periodontally healthy chronic periodontitis and chronic periodontitis patients with type 2 diabetes mellitus. J Investig Clin Dent. 2019;10(3):e12420. 10.1111/jicd.12420. 31172690 10.1111/jicd.12420

[8] Nikbin A, Bayani M, Jenabian N, Khafri S, Motallebnejad M. Oral health-related quality of life in diabetic patients: comparison of the Persian version of geriatric oral health assessment index and oral health impact profile: A descriptive-analytic study. J Diabetes Metab Disord. 2014;13(1):32. 10.1186/2251-6581-13-32. 24495383 10.1186/2251-6581-13-32 PMC4015305

[9] Khalifa N, Rahman B, Gaintantzopoulou MD, Al-Amad S, Awad MM. Oral health status and oral health-related quality of life among patients with type 2 diabetes mellitus in the united Arab emirates: a matched case-control study. Health Qual Life Outcomes. 2020;18(1):182. 10.1186/s12955-020-01418-9. 32539861 10.1186/s12955-020-01418-9 PMC7294625

[10] Atchison KA, Dolan TA. Development of the geriatric oral health assessment index. J Dent Educ. 1990;54(11):680–7. 2229624

[11] Slade GD, Spencer AJ. Development and evaluation of the oral health impact profile. Community Dent Health. 1994;11:03–11. 8193981

[12] Locker D, Miller Y. Evaluation of subjective oral health status indicators. J Public Health Dent. 1994;54(3):167–76. 10.1111/j.1752-7325.1994.tb01209.x. 7932353 10.1111/j.1752-7325.1994.tb01209.x

[13] Leao A, Sheiham A. The development of a socio-dental measure of dental impacts on daily living. Community Dent Health. 1996;13(1):22–6. 8634892

[14] Slade GD. Derivation and validation of a short-form oral health impact profile. Community Dent Oral Epidemiol. 1997;25(4):284–90. 10.1111/j.1600-0528.1997.tb00941.x. 9332805 10.1111/j.1600-0528.1997.tb00941.x

[15] Tabesh A, Mahmood M, Sirous S. Oral health-related quality of life and xerostomia in type 2 diabetic patients. Dent Med Probl. 2023;60(2):227–31. 10.17219/dmp/147754. 37366296 10.17219/dmp/147754

[16] Tonetti MS, Greenwell H, Kornman KS. Staging and grading of periodontitis: framework and proposal of a new classification and case definition [published correction appears in J periodontol. 2018;89(12):1475. Doi: 10.1002/jper.10239]. J Periodontol. 2018;89(Suppl 1):S159–72. 10.1002/JPER.18-0006. 29926952 10.1002/JPER.18-0006

[17] Loe H, Silness J. Periodontal disease in pregnancy. I. Prevalence and severity. Acta Odontol Scand. 1963;21:533–51. 10.3109/00016356309011240. 14121956 10.3109/00016356309011240

[18] Quigley GA, Hein JW. Comparative cleansing efficiency of manual and power brushing. J Am Dent Assoc. 1962;65:26–9. 10.14219/jada.archive.1962.0184. 14489483 10.14219/jada.archive.1962.0184

[19] Saxer UP, Mühlemann HR. Motivation und aufklärung [Motivation and education]. SSO Schweiz Monatsschr Zahnheilkd. 1975;85(9):905–19. German. 1059253

[20] Thomson WM, Chalmers JM, Spencer AJ, Williams SM. The Xerostomia inventory: a multi-item approach to measuring dry mouth. Community Dent Health. 1999;16(1):12–7. 10697349

[21] Thomson WM, Williams SM. Further testing of the Xerostomia inventory. Oral Surg Oral Med Oral Pathol Oral Radiol Endod. 2000;89(1):46–50. 10.1016/s1079-2104(00)80013-x. 10630941 10.1016/s1079-2104(00)80013-x

[22] Bots CP, Brand HS, Veerman EC, Korevaar JC, Valentijn-Benz M, Bezemer PD, Valentijn RM, Vos PF, Bijlsma JA, ter Wee PM, Van Amerongen BM, Nieuw Amerongen AV. Chewing gum and a saliva substitute alleviate thirst and Xerostomia in patients on haemodialysis. Nephrol Dial Transpl. 2005;20(3):578–84. 10.1093/ndt/gfh675. Epub 2005 Jan 21. 10.1093/ndt/gfh675 15665029

[23] Locker D, Quiñonez C. Functional and psychosocial impacts of oral disorders in Canadian adults: a National population survey. J Can Dent Assoc. 2009;75(7):521. 19744362

[24] Mauri-Obradors E, Estrugo-Devesa A, Jané-Salas E, Viñas M, López-López J. Oral manifestations of diabetes mellitus. A systematic review. Med Oral Patol Oral Cir Bucal. 2017;22(5):e586–94. 10.4317/medoral.21655. 28809366 10.4317/medoral.21655 PMC5694181

[25] Ryan ME, Carnu O, Kamer A. The influence of diabetes on the periodontal tissues. J Am Dent Assoc. 2003;134:34S-40S. 10.14219/jada.archive.2003.0370. 18196671 10.14219/jada.archive.2003.0370

[26] Manfredi M, McCullough MJ, Vescovi P, Al-Kaarawi ZM, Porter SR. Update on diabetes mellitus and related oral diseases. Oral Dis. 2004;10(4):187–200. 10.1111/j.1601-0825.2004.01019.x. 15196139 10.1111/j.1601-0825.2004.01019.x

[27] Verhulst MJ, Teeuw WJ, Gerdes VE, Loos BG. Self-reported oral health and quality of life in patients with type 2 diabetes mellitus in primary care: a multi-center cross-sectional study. Diabetes Metab Syndr Obes. 2019;12:883–99. 10.2147/DMSO.S207087. 31354324 10.2147/DMSO.S207087 PMC6590843

[28] Molania T, Alimohammadi M, Akha O, Mousavi J, Razvini R, Salehi M. The effect of Xerostomia and hyposalivation on the quality of life of patients with type II diabetes mellitus. Electron Physician. 2017;9(11):5814–9. 10.19082/5814. 29403624 10.19082/5814 PMC5783133

[29] Niklander S, Veas L, Barrera C, Fuentes F, Chiappini G, Marshall M. Risk factors, hyposalivation and impact of xerostomia on oral health-related quality of life. Braz Oral Res. 2017;31:e14 ,1–9. 10.1590/1807-3107BOR-2017.vol31.0014. 10.1590/1807-3107BOR-2017.vol31.0014 28099580

[30] Botelho J, Machado V, Proença L, Oliveira MJ, Cavacas MA, Amaro L, Águas A, Mendes JJ. Perceived xerostomia, stress and periodontal status impact on elderly oral health-related quality of life: findings from a cross-sectional survey. BMC Oral Health. 2020;20(1):199. 10.1186/s12903-020-01183-7. 32650751 10.1186/s12903-020-01183-7 PMC7350690

[31] Preshaw PM, Alba AL, Herrera D, et al. Periodontitis and diabetes: a two-way relationship. Diabetologia. 2012;55(1):21–31. 10.1007/s00125-011-2342-y. 22057194 10.1007/s00125-011-2342-y PMC3228943

